# Exogenous chemically-driven electromagnets†

**DOI:** 10.1039/d5sc00911a

**Published:** 2025-06-16

**Authors:** Cara Lozon, Antoine Cornet, Stéphane Reculusa, Patrick Garrigue, Alexander Kuhn, Gerardo Salinas

**Affiliations:** a Univ. Bordeaux, CNRS UMR 5255, Bordeaux INP, Site ENSMAC 33607 Pessac France gerardo.salinassanchez@enscbp.fr

## Abstract

Magnetically-driven dynamic systems have gained considerable attention in multiple applications ranging from cargo delivery to environmental remediation. However, they commonly require ferromagnetic components or sophisticated electromagnetic equipment. In this work we take advantage of the synergy between exogenous bipolar electrochemistry and the classic geometry of a solenoid in order to design an externally driven chemo-electromagnet. By wirelessly triggering redox reactions at each extremity of a solenoid-shaped swimmer, the generated electric current follows the helical path of the coil, thus generating a concentric magnetic field in its center. Such an externally induced redox current generates magnetic fields in the range of μT which are proportional to the applied electric field. The on-board chemically induced magnetic dipole allows the swimmers to perform rotational motion in the presence of an external magnetic field, without the use of traditional ferromagnetic materials. Additionally, when exposing these devices to alternating electric and magnetic fields, well-defined oscillatory motion is produced, demonstrating the efficient electromagnetic control of the dynamic displacement. This opens up novel and, so far, unexplored possibilities for localized chemical conversion *via* magnetically-driven “chemistry on-the-fly”.

## Abstract 2

Externally driven chemo-electromagnets were designed by coupling bipolar electrochemistry and the geometry of a solenoid. The chemically induced magnetic dipole allows control of the dynamic displacement, without the use of ferromagnetic materials.

## Graphical abstract

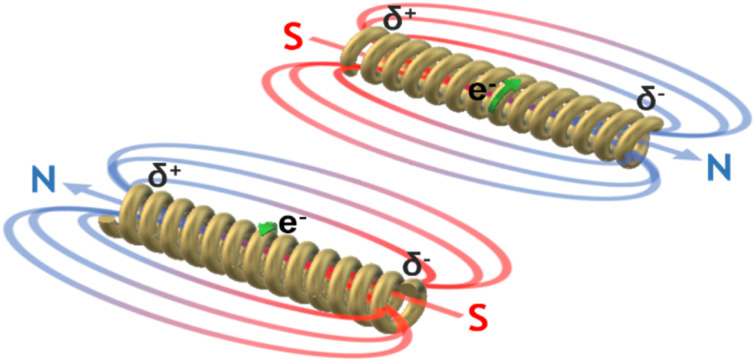

## Introduction

Magnetically driven dynamic systems have attracted significant attention across various fields of science,^1–3^ including cargo delivery,^4,5^ sensing,^6,7^ and environmental remediation.^8–10^ These systems typically rely on propulsion triggered by a magnetic pull mechanism or rotational and undulating motions.^11–13^ While being very efficient, such devices usually require ferromagnetic components, such as nickel (Ni), iron (Fe) or cobalt (Co), coupled with a precise control of external magnetic fields *via* sophisticated electromagnetic equipment.^14–17^ Additionally, ferromagnetic materials can cause biocompatibility issues when used in medical or biological applications. Moreover, coating ferromagnetic materials with biocompatible layers adds complexity to their design. Therefore, the development of approaches that induce an on-board magnetic field without relying on ferromagnetic components is highly desirable.

A promising approach is to exploit the conventional working principle of a solenoid-shaped electromagnet.^18^ According to Ampere's law, a current-carrying coiled wire generates a concentrated magnetic field along its axis. The strength of this magnetic field can be tuned by changing the number of loops and the magnitude of the current flowing through the solenoid.^19,20^ Such devices are crucial components in many everyday devices, including electric motors, generators, and alternators. However, they typically rely on the direct electrical connection to an external power source in order to maintain the current flow, which limits their applicability, particularly in environments where electrical connections are impractical or undesirable.

In this context, bipolar electrochemistry (BE) has emerged as a powerful tool for inducing in a wireless manner an electric current across objects.^21–23^ When a polarization potential difference (Δ*V*) is generated across a conducting object, acting as bipolar electrode (BPE), redox reactions occur at both extremities of the device once Δ*V* exceeds the thermodynamic threshold potential (Δ*V*_min_) of the involved redox reactions. The ability to induce an asymmetric polarization within a single object makes BE highly attractive for multiple applications ranging from surface modification^24,25^ and electroorganic synthesis^26–28^ to motion generation^29,30^ and electroanalysis.^31,32^ Interestingly, such a Δ*V* can be induced either by coupling thermodynamically spontaneous redox reactions (endogenous, Δ*G* < 0) or by applying a high enough external electric field (*ε*) powering non-spontaneous reactions (exogenous, Δ*G* > 0).^33–35^ Recently the concept of endogenous BE has been exploited to induced an on-board magnetic field in coiled-shaped Janus BPEs.^36^ The thermodynamically spontaneous redox reactions induce a helical flow of electrons from the anodic to the cathodic extremity of the solenoid-shaped device, generating a concentrated magnetic field inside the coil. This concept presents a novel paradigm for wireless electromagnetism, where the current is driven not by direct electrical connections but by redox chemistry. A significant advantage is that the chemically induced magnetic field is generated as long as the solenoid interacts with its chemical environment. However, this type of system operates continuously once placed in solution, as the redox processes are driven by thermodynamically favored reactions. As a result, the magnitude of the magnetic field is limited by the swimmer's inherent redox chemistry.

In contrast to endogenous systems, exogenous bipolar electrochemistry allows for the external triggering of non-spontaneous redox reactions (Δ*G* > 0) by fine-tuning the applied electric field (*ε*). In this work, the synergy between exogenous BE and the classic solenoid shape was exploited to design a wireless, externally driven chemo-electromagnet. By exploiting redox reactions at the extremities of an Au/Ti solenoid-shaped swimmer, the ability to generate an on-board magnetic field is demonstrated. In principle, the system can behave as a switchable chemo-electromagnet, where the magnetic field can be easily turned on-off by modulating the external electric field. This feature provides precise control over the swimmer's magnetic field, expanding its potential applications in fields requiring magnetic manipulation. This on-board chemically induced magnetic field enables simultaneous control of the swimmer's dynamics and the electrochemical transformation of an analyte of interest. Therefore, it is possible to envision utilizing these devices, for example, in electro-organic synthesis as dynamic macro- and micro-reactors. In this instance, the *in situ* transformation would enhance mass transport, addressing one of the main limitations in electrocatalysis.

## Results and discussion

**Scheme 1:**
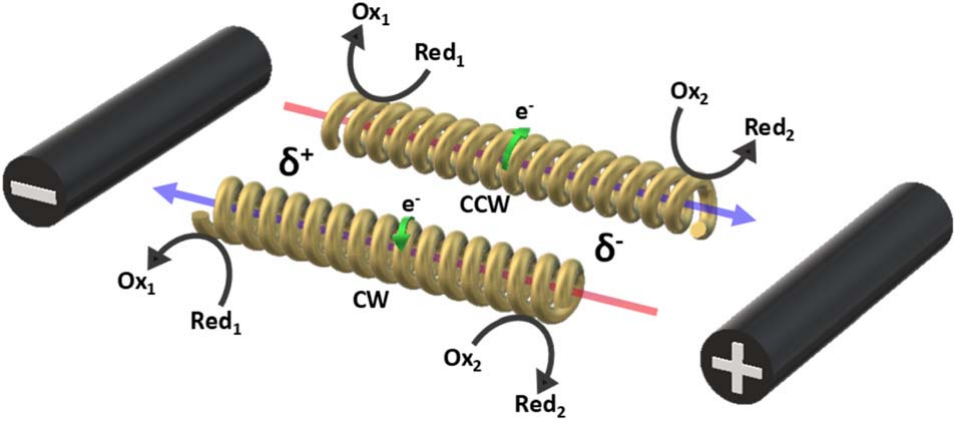
Illustration of the current produced by the wirelessly triggered redox reactions taking place at the extremities of a clockwise or anticlockwise solenoid, with a representation of the currents produced by the chemical reactions and the exogenous chemically-induced magnetic field. The blue and red colored arrows represent the north and south pole of the generated magnetic fields, respectively.

The concept of exogenous chemo-electromagnets is based on the synergetic coupling of two principles: the externally-driven electric current passing through a BPE and the features of a classic solenoid electromagnet (Scheme 1). Briefly, by applying a high enough external *ε* in the presence of electroactive species, an oxidation and a reduction reaction occur at the anodic and cathodic extremities of the solenoid-shaped BPE (Scheme 1). These redox reactions are accompanied by a flux of electrons which follows the helical path of the coil-shaped object, generating a magnetic field according to the principles of electromagnetism. Furthermore, the orientation of the magnetic field is intimately related to the direction of the winding, hence a clockwise orientation induces the north and south poles at the anode and cathode of the device, whereas the anticlockwise winding produces the opposite polarity (Scheme 1). The Au/Ti solenoid-shaped swimmer was designed by tightly wrapping a Ti wire (*ø* = 0.25 mm) around a glass rod (*ø* = 0.6 mm) to form the solenoid. Titanium was chosen as a core material due to its relatively low density, allowing the coil to stay at the air–liquid interface. Subsequently, the device was removed from the rod and trimmed in order to obtain a solenoid of 16 loops (*l* = 5.7 mm). After this, the object was etched with HF (10%) to remove an eventually present oxide layer and then covered with a thin layer of Au in order to avoid the possible formation of a passivation layer of TiO_2_ during the bipolar electrochemistry experiments. The final composition of the Au/Ti solenoid-shaped swimmer was studied by energy dispersive X-ray spectrometry (EDX) (Fig. S1†). EDX analysis exhibits the characteristic signals of Ti (Ti-Kα) and Au (Au-Kα) (Fig. S1†) whereas the optical pictures exhibit a rather homogeneous deposit of Au on the surface of the Ti coil (inset Fig. S1†), confirming the surface modification.

**Fig. 1:**
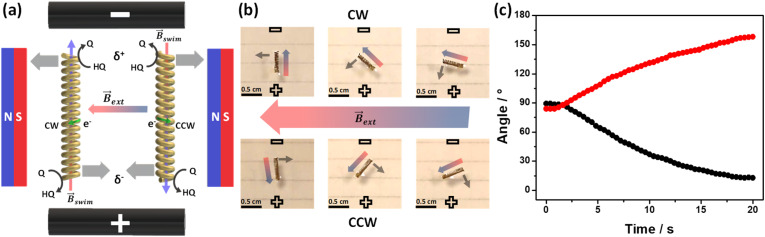
(a) Schematic illustration and (b) optical pictures of the rotational displacement of a clockwise and anticlockwise coiled exogenous chemo-electromagnet in the presence of an external magnetic field, with a representation of the chemical reactions, the direction of the associated electron flow, the orientation of the induced and external magnetic fields, the external electric field, the torque force and the trajectory of the rotation. The blue and red color represent the north and south pole of the magnetic fields, respectively. Global time of the experiment was 20 seconds. (c) Average angular progression as a function of time, measured during the displacement of a clockwise (black dots) and anticlockwise (red dots) Au/Ti solenoid moving at the air/water interface of a 5 mM LiClO_4_/10 μM DBS solution containing a 1 : 1 ratio of HQ/Q (10 : 10 mM) in the presence of an external magnetic field and a constant applied electric field (4.2 V cm^−1^).

After this characterization, the possible generation of an electromagnetic field *via* externally driven redox reactions was evaluated. As stated above, when a high enough electric field is applied, redox reactions are triggered at each extremity of the solenoid-shaped device. Hence electrons are flowing across the helical path of the object from the anode to the cathode. Considering the principles of a conventional current-carrying solenoid wire, it is possible to assume that the asymmetrically polarized coil can induce its own concentrated magnetic field. In order to test this hypothesis, the dynamic behaviour of the externally-driven chemo-electromagnets was evaluated in the presence of an external magnetic field. For this, the solenoid was placed at the air/water interface of a 5 mM LiClO_4_, 10 μM DBS solution, containing hydroquinone (HQ) and quinone (Q) in an equimolar ratio (10 mM). The HQ/Q redox pair was chosen as model electroactive probe, since it presents a relatively small Δ*V* (114 mV) (Fig. S2†). In addition, both Q and HQ are organic, diamagnetic molecules, meaning that they do not interact with the external magnetic field. This is crucial for demonstrating the operation principles of the device in the absence of ferromagnetic components. A homogeneous external magnetic field 
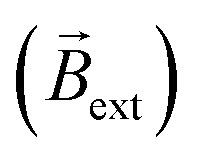
 was generated by placing two identical FeNdB permanent magnets with their poles aligned (*B*_surface_ ≈ 250 mT, *A* = 8 cm^2^), at the extremities of the bipolar cell at a distance of 12 cm (Fig. 1a). Under this configuration, a magnetic field of 15 mT was generated at the center of the bipolar cell (Fig. S3†). The same experimental setup was used for all the measurements unless otherwise indicated. When applying a high enough *ε*, the oxidation and reduction of hydroquinone and quinone take place at the respective extremities of the coil (eqn (1)).

1 HQ ⇌ Q + 2H^+^ + 2e^−^

These reactions produce a helical flow of electrons along the solenoid, which in turn induces a concentric magnetic field inside the coil-shaped object 
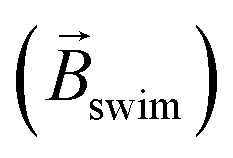
. In theory, the presence of a 
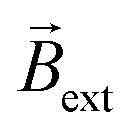
 leads to the electromagnetic self-alignment of the device, which translates in a rotational displacement (Fig. 1a). As it can be seen, by applying a constant electric field (4.2 V s^−1^) and in the presence of 
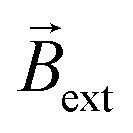
, both the clockwise (CW) and counterclockwise (CCW) Au/Ti solenoids exhibit a rotational displacement in order to align 
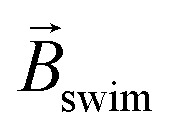
 and 
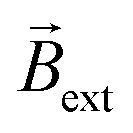
 (Fig. 1b and Video S1†). Such an electromagnetic alignment is characteristic of the polarized solenoid since an Au wire (*l* = 0.6 cm), acting as BPE, presents only a very slow linear drift under the same experimental conditions (Video S2†). In order to corroborate the synergy effect between the external electric and the magnetic field, the dynamic behaviour of the solenoid-shaped BPE was evaluated in the presence of exclusively one external perturbation (Video S3†). In the absence of 
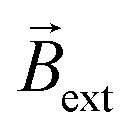
, the BPE exhibits a rather slow linear motion, mainly toward the feeder cathode, probably due to an electrophoretic propulsion mechanism (Video S3† left panel). On the contrary, in the absence of *ε*, the swimmer presents a random motion (Video S3† right panel). These results confirm that the externally polarized solenoid generates a magnetic dipole, which interacts with 
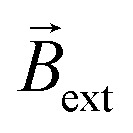
 to trigger rotational motion. It is important to emphasise that this rotational motion is not driven by the Laplace force. In a uniform magnetic field, the net force on the solenoid is zero due to the cancelling of the Lorentz forces on opposite sides of each loop (Fig. S4†). However, the solenoid experiences a torque due to its chemically induced magnetic dipole moment, given by,2
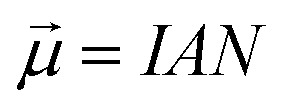
where 
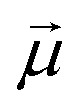
 is the magnetic dipole moment of the solenoid, *I* is the current, *A* is the cross-sectional area and *N* is the number of windings. This magnetic dipole moment interacts with the external magnetic field, producing a torque given by3
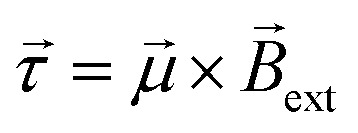
and is strongest when 
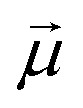
 is orthogonal to 
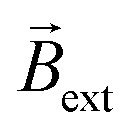
 and parallel to the external electric field.

However, as the solenoid-shaped BPE rotates, a decrease of the angular speed was observed, which leads to a maximum rotation angle of approximately 70° (Fig. 1c). According to the principles of BE, the Δ*V* induced by a given electric field is a function of the angle (*α*) formed between the electric field and the object main axis (eqn (4)).

4 Δ*V* = *εl* cos *α*

Hence, when the coil-shaped BPE rotates, Δ*V* consequently decreases as a function of the cosine of *α*, and thus, the intensity of electrochemically generated current passing through the solenoid decreases. Since the magnitude of *B*_swim_ is directly related to this current, the externally triggered chemo-electromagnetic field weakens during rotation. This was analysed in more detail by plotting the angular acceleration and the theoretical Δ*V* as a function of the angle of rotation (Fig. S5†). As it can be seen, the maximum angular acceleration (≈5° s^−2^) is found for *α* = 0°, that is when the main axis of the BPE is parallel to the orientation of *ε*, thus the highest theoretical Δ*V* is induced along the coil-shaped BPE. Then a steep decrease of the angular acceleration is observed. Nonetheless the BPE exhibits a continuous displacement reaching a maximum of *α* = 80°, where the current and the related induced magnetic field are no longer sufficient to be affected by 
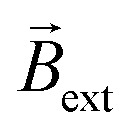
. To provide further evidence of the relation between the magnitude of *B*_swim_ and the angle of rotation, the current passing through the solenoid-shaped BPE was evaluated (Fig. S6a†). For this experiment, two separate Au/Ti coils composed of 8 loops each, were connected to an ammeter and placed in the bipolar cell containing a 5 mM LiClO_4_, 10 μM DBS solution, in the presence of HQ/Q in an equimolar ratio (10 mM). The measurement was performed by changing manually the angle (*α*) formed between the electric field and the BPE main axis. As expected, under a constant *ε* value (4.2 V cm^−1−1^) a maximum current (≈0.2 mA) is produced when *α* = 0° (Fig. S6a,† green dots), which decreases as a function of the angle of rotation. This drop of current correlates with the profile of the Δ*V* calculated with eqn (3) (Fig. S6† grey line). From these current values, it is possible to estimate the magnetic field generated by the externally driven redox reactions by means of eqn (5).5
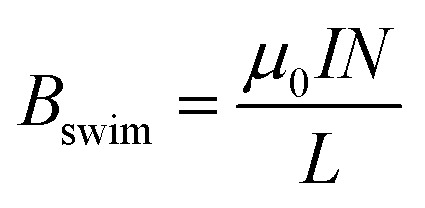
where *B*_swim_ is the magnetic field generated by the polarized solenoid, *μ*_0_ is the permeability of free space (4π × 10^−7^ T m A^−1^), *N* is the number of turns, *L* is the length of the solenoid, and *I* is the current passing through the coil-shaped device. A maximum magnetic field value of around 0.8 μT was obtained (Fig. S6b†). A steep decrease of *B*_swim_ as a function of the rotation angle is observed, in agreement with the theoretical Δ*V* and the angular acceleration estimations.

**Fig. 2:**
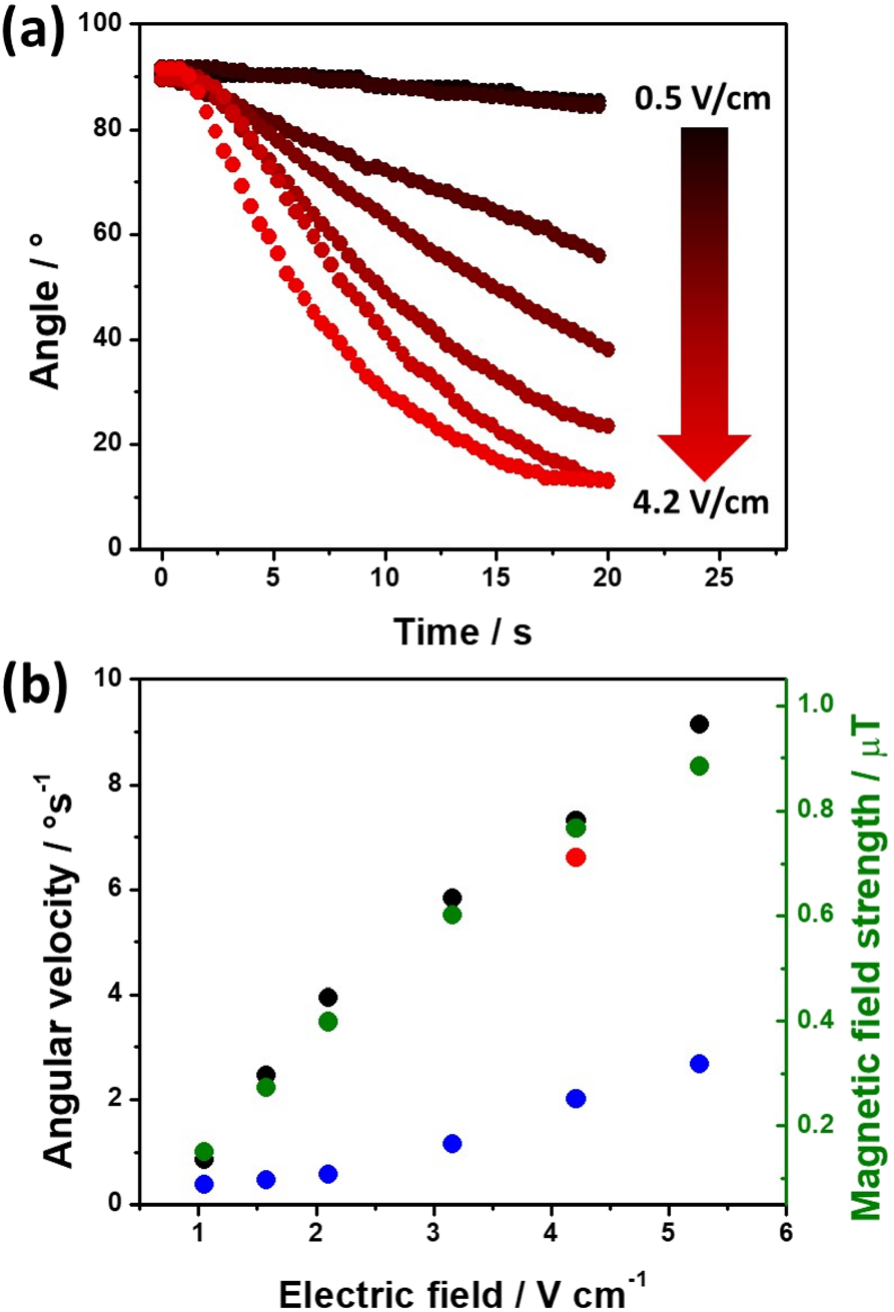
(a) Angular progression as a function of time observed during the rotation of a clockwise Au/Ti solenoid moving at the air/water interface of a 5 mM LiClO_4_/10 μM DBS solution containing a 1 : 1 ratio of HQ/Q (10 : 10 mM) in the presence of an external magnetic field, for different applied electric fields (indicated in the figure). (b) Maximum angular velocity of the swimmer (left axis black dots) as a function of the applied electric field and the average *B*_swim_ value estimated from amperometric measurements (right axis green dots). The red dot refers to a measurement carried out with the anticlockwise Au/Ti solenoid. The blue dots refer to the maximum angular velocity of the swimmer as a function of the applied electric field for the oxygen and hydrogen evolution reactions.

After this set of experiments, corroborating the external induction of a chemo-electromagnetic field, the influence of the electric field on the magnitude of *B*_swim_ within the coil and its concomitant angular displacement has been evaluated. According to the principles of BE, a decrease of the applied *ε* leads to a lower Δ*V* across the BPE (eqn (4)). This has an impact on the kinetics of the redox reactions, which in turn, due to the solenoidal shape of the BPE, will influence the magnitude of *B*_swim_. This effect was investigated by using electric field values ranging from 0.5 to 5.3 V cm^−1^. Under these conditions, in theory, it is possible to induce a sufficient Δ*V* (between 0.28 and 3.0 V), triggering the corresponding redox reactions. However, at relatively low *ε* values (below 0.5 V cm^−1^) no rotational displacement was observed (Video S4† left panel), which is attributed to a too low Δ*V* (≈0.28 V) induced across the BPE. Above this *ε* value, the redox reactions take place, generating the chemo-electromagnetic field within the solenoid. This leads to the characteristic rotation related to the self-alignment between 
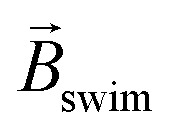
 and 
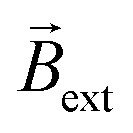
 (Video S4† center and right panels). Fig. 2a presents the angular transients obtained by applying different electric field values. As it can be seen, as *ε* increases, a steeper variation of the rotation angle as a function of time is produced. From the slope of the linear region of the angular displacement, the angular velocity was evaluated. A rather linear correlation between the angular velocity and *ε* was obtained in the range between 0.9° s^−1^ and 9.1° s^−1^ (Fig. 2b black dots). In order to correlate these results with the approximate magnitude of the induced chemo-electromagnetic field, the currents passing through the coil-shaped BPE were once again measured. For electric fields from 0.5 to 5.3 V cm^−1^, the calculated *B*_swim_ gradually decreases as a function of the angle of rotation (Fig. S6b†). However, plotting the maximum *B*_swim_, that is when the main axis of the BPE is parallel to the electric field, for each applied *ε* value, a linear tendency in the range between 0.15 μT and 0.9 μT was obtained (Fig. 2b green dots). As expected, both profiles, the angular velocity and *B*_swim_, overlap, presenting a linear dependence as a function of the external electric field. It is important to highlight that similar angular velocity values were obtained for the CW and CCW devices (Fig. 2b, red dot). These results allow us to conclude that the angular displacement of the swimmer in the presence of 
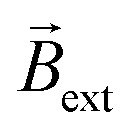
 is a direct consequence of the solenoidal architecture of the BPE and the externally-driven polarization, that produces a wireless non-ferromagnetic exogenous chemo-electromagnet. Alternatively, according to the principles of BE, it is possible to fine-tune Δ*V* across the solenoid-shaped BPE, and its concomitant induced magnetic field, by changing the length of the device. Changes in the length of the BPE can be achieved by increasing the number of coils of the solenoid. This will directly impact the magnitude of the chemo-electromagnetic field in agreement with eqn (4). Au/Ti solenoids composed by 8, 16 and 32 coils were designed and their dynamic behavior was evaluated independently in the presence of a constant electric and magnetic field (4.2 V s^−1^ and 15 mT, respectively). As expected, all three swimmers rotate in order to self-align their internal electromagnetic field with 
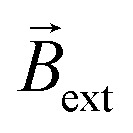
 (Video S5†). Unexpectedly, a decrease of the angular velocity was obtained for the larger coil-shaped BPE (36 loops) (Fig. S7† top, black dots). Although, in theory the polarization potential difference increases linearly with the length of the BPE (and the number of coils) (Fig. S7† bottom), other physical factors may hinder the dynamic displacement of the rotor, *e.g.* friction at the water/air interface or the weight of the solenoid. By plotting the weight as a function of the number of coils a direct correlation with the Δ*V* is obtained (Fig. S7†). However, as the weight increases, the induced electrochemical driving force is overcompensated by the additional torque required to surpass the increase of friction and momentum associated with larger objects. This is reflected by the decrease of angular speed for the solenoid-shaped BPE composed of 32 coils (Fig. S7†).

Furthermore, in order to illustrate the general validity of the concept, the dynamic behaviour of the externally-driven chemo-electromagnets was evaluated by using an alternative redox system, *e.g.* hydrogen and oxygen evolution reactions (HER and OER, respectively). For this, the solenoid was placed at the air/water interface of a 5 mM LiClO_4_, 10 μM DBS solution, in the absence of the HQ/Q redox couple. HER and OER reactions were chosen as alternative electroactive systems, since they present a rather larger Δ*V* on Au (2.4 V) (Fig. S2†). Once again, when applying a high enough *ε*, the oxidation and reduction of water take place at the extremities of the coil (eqn (6) and (7), respectively), producing the helical flux of electrons that induces the chemo-electromagnetic field.

6 2H_2_O → 4H^+^ + O_2_ + 4e^−^

7 2H^+^ + 2e^−^ → H_2_

However, due to the considerably larger Δ*V* value required to trigger these redox reactions, a substantial decrease of the angular velocity is expected in the same range of applied electric field values (0.5 to 5.3 V cm^−1^). As it can be seen, at low *ε* values (below 2.1 V cm^−1^) there is not enough driving force (Δ*V* < 1.2 V) to trigger the OER and HER at the anode and cathode of the coil, thus no rotational displacement was observed (Video S6† left and center panels). Above this threshold electric field value, the redox reactions take place, inducing the chemo-electromagnetic field 
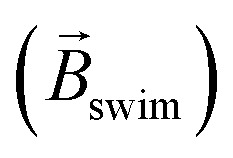
, which slowly self-aligns with 
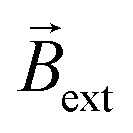
 (Video S6† right panel). For even higher *ε* values, a linear increase of the angular velocity as a function of the applied *ε* was observed (Fig. 2b, blue dots). Moreover, this plot provides evidence for the impact of the thermodynamic nature of the redox probes on the onset electric field (*ε*_onset_) required to induce 
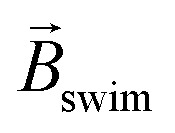
 (Fig. 2b). Thus, redox pairs with rather small Δ*V* values (114 mV), such as HQ/Q, induce motion at low *ε*_onset_ values (0.5 V cm^−1^), whereas for electroactive systems with a large thermodynamic potential difference (Δ*V* = 2.4 V) require higher *ε*_onset_ (2.1 V cm^−1^). This opens up new opportunities for the straightforward screening of electroactive systems, using the angular velocity as a dynamic readout of chemical information.

**Fig. 3:**
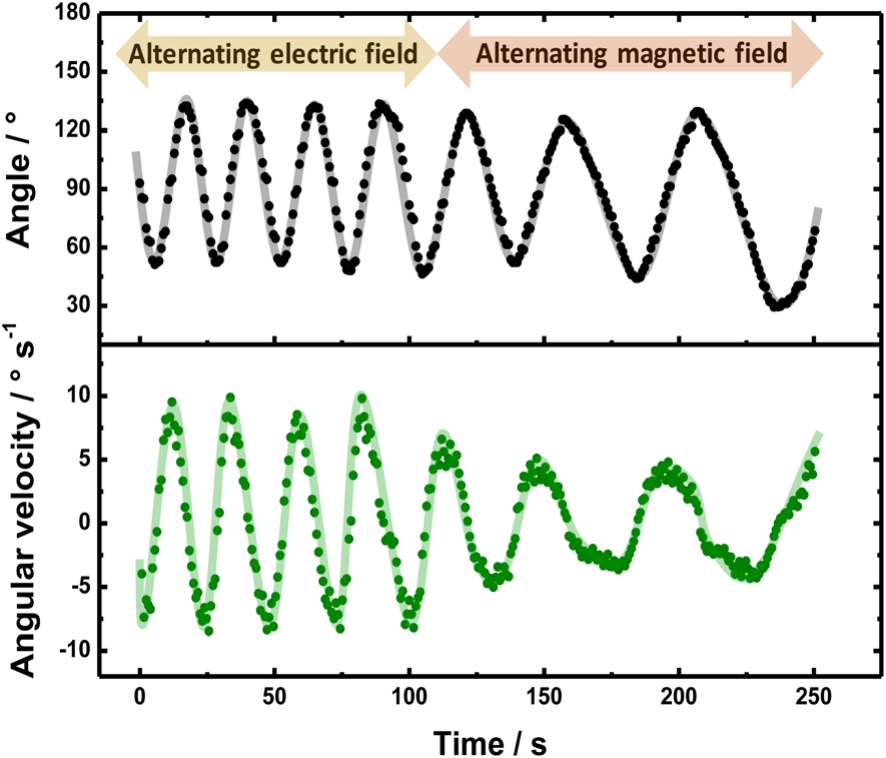
(Top) Angular progression and (bottom) angular velocity as a function of time observed during the rotation of a counter clockwise Au/Ti solenoid moving at the air/water interface of a 5 mM LiClO_4_/10 μM DBS solution containing a 1 : 1 ratio of HQ/Q (10 : 10 mM) in the presence of an alternating external electric (3.15 V cm^−1^) and magnetic field (indicted in the figure).

Finally, after demonstrating the induction of magnetic fields by BE, the possible electric and magnetic control of the dynamic displacement has been evaluated. In theory when using a high enough alternating electric field, the direction of the current passing through the solenoid-shaped BPE changes according to the orientation of the external *ε* (Fig. S8a†) This leads to a periodic change of the orientation of the induced magnetic field within the coil, which in the presence of a homogeneous 
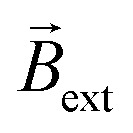
 leads to a oscillatory rotation. This type of electromagnetic alignment can also be generated by an alternating external magnetic field in the presence of an external electric field with constant orientation (Fig. S8b†). With every reversal of the external magnetic field, the devices rotates in order to reach an alignment between 
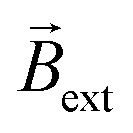
 and 
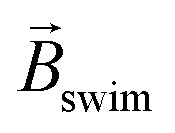
. In a first test, with a constant external magnetic field orientation, an alternating *ε* (3.15 V cm^−1^), with a frequency of 0.1 Hz, was applied. Subsequently, the orientation of 
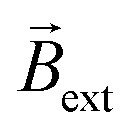
 was manually reversed, in the presence of a constant electric field (3.15 V cm^−1^). This kind of perturbation scheme allows evaluating the electric and magnetic control of the dynamics in a single measurement. The angular progression and angular velocity were plotted as a function of time for a more quantitative analysis (Fig. 3). As it can be seen, initially under the influence of the alternating *ε*, the CCW solenoid exhibits an oscillatory displacement due to the continuous change of the orientation of its internal magnetic field in order to self-align with 
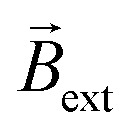
 (Video S5† left panel). The periodicity of the displacement is reflected by the ±40° rotation, from the initial position (90°) of the device (Fig. 3 top, black dots). Furthermore, for the angular velocity transient, the maximum value is reached when the solenoid is perfectly parallel to the external electric field (Fig. 3 bottom, green dots), which is in agreement with eqn (3). When alternating the external magnetic field in a constant electric field, a similar oscillatory pattern of the motion was observed (Video S7†). These periodic changes in torque, induced by alternating electric and magnetic fields, produced the observed oscillations in both angular displacement and velocity (Fig. 3). Overall, the behavior of the solenoid swimmer under these alternating fields demonstrates its responsiveness to external stimuli. It is important to point out that when comparing the right and left panel of Video S7,† a specular rotational trajectory is obtained for the CW coiled solenoid, evidencing the control of the electromagnetic field polarity by the sense of the windings.

## Conclusions

In summary, an externally driven chemo-electromagnet was designed by coupling the features of exogenous BE with solenoid-type conducting objects. This unique and efficient approach allows generating magnetic fields in the range of μT by taking advantage of the current produced by the stimulated redox reactions. Such an on-board magnetic dipole enables the efficient control of rotational motion in the presence of an external magnetic field, without the use of traditional ferromagnetic materials. A direct correlation between the angular velocity and the intensity of the chemically induced magnetic field was observed. These devices exhibit a well-controlled oscillatory motion when exposed to alternating electric and magnetic fields. In fact, in a first order of approximation the periodicity of the swimmer's motion can be easily fine-tuned by modifying the magnitude of the external perturbation. Such a feature enables the possible use of these devices for the design of mechanical oscillators or microfluidic mixers, where periodic motion allows overcoming mass transport limitations. Although this is a proof-of-concept work, it is possible to assume that this concept is generic, hence different electroactive systems can be used as electron source. Thus, this feature opens up novel and, so far, unexplored possibilities for localized chemical conversion, such as in “chemistry on-the-fly” systems,^37–39^ since the chemo-electromagnets act as externally propelled reactors. Thus, we envisage the development of different redox-based solenoids for multiple applications ranging from sensing and electro-organic synthesis to environmental remediation.

## Author contributions

C. L. and A. C. performed the electrochemical and dynamic experiments and analysed the data. S. R. and P. G. performed device characterization measurements. A. K. and G. S. directed the study and designed the research. All authors prepared the manuscript and approved the final version.

## Conflicts of interest

There are no conflicts to declare.

## Supplementary Material

SC-016-D5SC00911A-s001

SC-016-D5SC00911A-s002

SC-016-D5SC00911A-s003

SC-016-D5SC00911A-s004

SC-016-D5SC00911A-s005

SC-016-D5SC00911A-s006

SC-016-D5SC00911A-s007

SC-016-D5SC00911A-s008

## Data Availability

Additional data can be obtained from the corresponding author upon request.
