# Supplementary material for: Exogenous chemically-driven electromagnets
Source: Chem Sci. 2025 Jun 16;16(29):13291–7. doi: 10.1039/d5sc00911a (PMC12184687; doi:10.1039/d5sc00911a)
Supplement: SC-016-D5SC00911A-s001 [file SC-016-D5SC00911A-s001.pdf]

Supplementary Information (SI) for Chemical Science.
This journal is © The Royal Society of Chemistry 2025

Exogenous chemically-driven electromagnets

Cara Lozon,[a] Antoine Cornet,[a] Stéphane Reculusa,[a] Patrick Garrigue,[a] Alexander Kuhn,[a] Gerardo Salinas*[a]

[a]Univ. Bordeaux, ISM, UMR 5255, Bordeaux INP Site ENSMAC, F 33607 Pessac, France

*Corresponding authors: gerardo.salinassanchez@enscbp.fr

Experimental details

Sodium dodecylbenzenesulfonate (Sigma-Aldrich, technical grade), $LiClO_4$ (Aldrich), Ti wire (Aldrich, 99.7%, ø = 0.25 mm), Hydroquinone (Aldrich, ≥ 99.5%) and p-Benzoquinone (Aldrich, ≥ 98%) were used as received. All solutions were prepared with deionized water (MilliQ Direct-Q, resistivity of 18.2 MΩ·cm at 25 °C). The solenoid devices were designed by winding a Ti wire tightly around a glass rod (d = 0.6 mm). Afterwards, the coil was removed from the rod and trimmed to obtain devices with different numbers of loops, 8, 16 and 32, generating solenoids of approximately 3.2 mm, 5.7 mm and 8.5 mm of length, respectively. Each Ti solenoid was etched with an HF solution (10%) for 5 min and afterwards modified by sputtering a thin gold layer (20 min, 35 mA). Energy-dispersive X-ray spectroscopy mappings were performed with a Vega3 Tescan 20.0 kV microscope. The electromagnetic self-alignment experiments were carried out in a plastic crystallizer (12 x 12 cm), acting as conventional bipolar cell, by placing the device at the air/water interface of a 10 µM sodium dodecylbenzenesulfonate, 5 mM $LiClO_4$ solution containing quinone/hydroquinone in an equimolar ratio (10 mM). The bipolar cell was composed by a reaction chamber separated from the feeder electrodes by two filter paper membranes. Graphite electrodes, acting as feeder electrodes, were positioned at the extremities of the cell at a distance of 9.5 cm. The external magnetic field was produced by placing two identical permanent FeNdB magnets ($B_{surface}$ ≈ 250 mT, A = 8 cm^2) parallel to each other with a separation of 12 cm. The electric and external magnetic fields were oriented orthogonal to each other (Figure 1a). The dynamic behavior of the swimmers was monitored by using a CCD camera (CANON EOS 70D, Objective Canon Macro Lens 100 mm 1:2.8). Video processing and tracking were performed with ImageJ software. Current measurements were carried out by using a Keithley electrometer/High resistance meter connected to a personal computer. For this, two separate Ti/Au coils, with eight loops each, were connected to the ammeter and then dipped into the corresponding working solution in the presence of different applied electric field values.

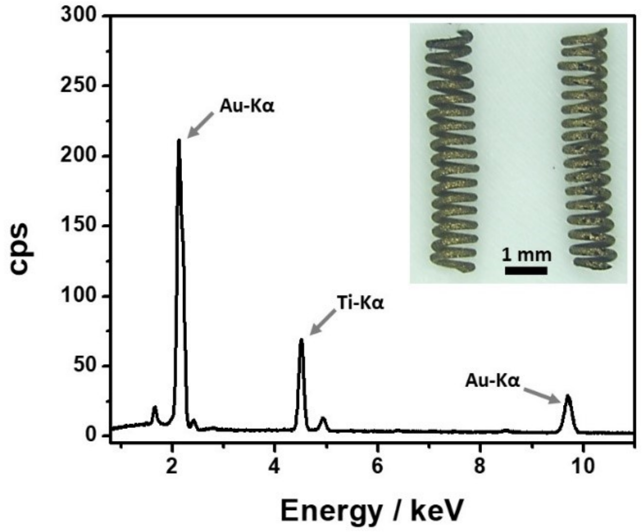

Figure S1. EDX signal around the emission peak of gold (Au Kα) and titanium (Ti Kα) along the surface of the swimmer coil. The insert with the optical picture shows the Au/Ti coiled swimmer ($ø_{Ti}$ = 250 µm).

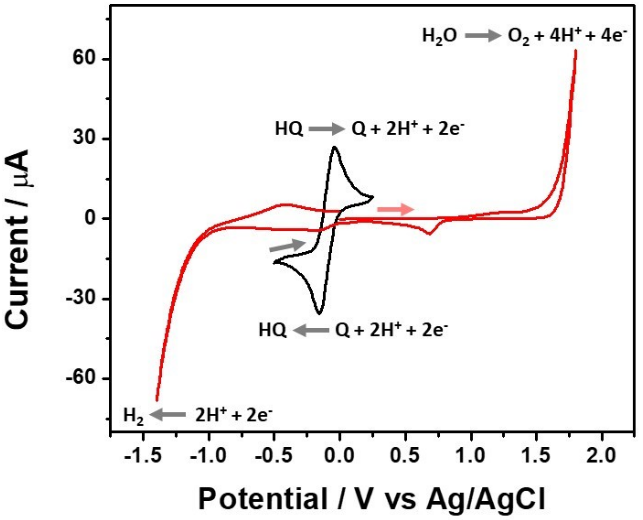

Figure S2. Potentiodynamic study with an Au disk electrode (ø = 3 mm) in a 0.1 M $LiClO_4$ solution in the absence (red line) and in the presence of a 1:1 ratio of HQ/Q (10:10 mM) (black line).

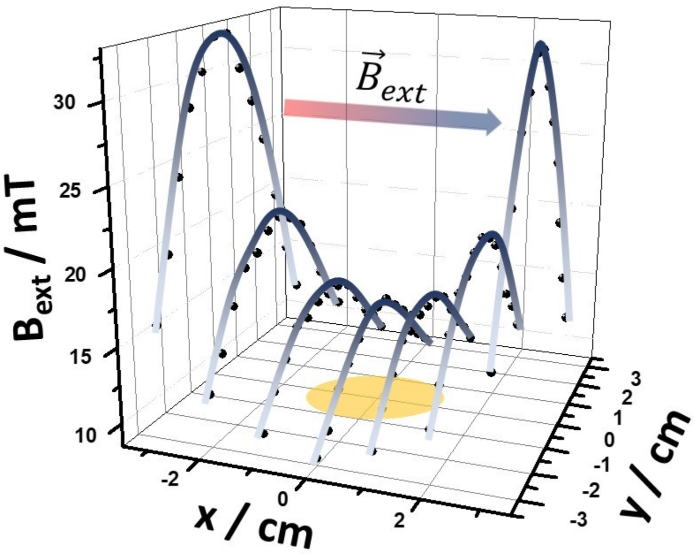

Figure S3. Plot of the distribution of the measured magnetic field between two identical permanent FeNdB magnets ($B_{surface}$ ≈ 250 mT, A = 8 cm^2). The yellow circle indicates the region where the rotation of the swimmer takes place. The blue/red colored arrow represents the orientation of the external magnetic field.

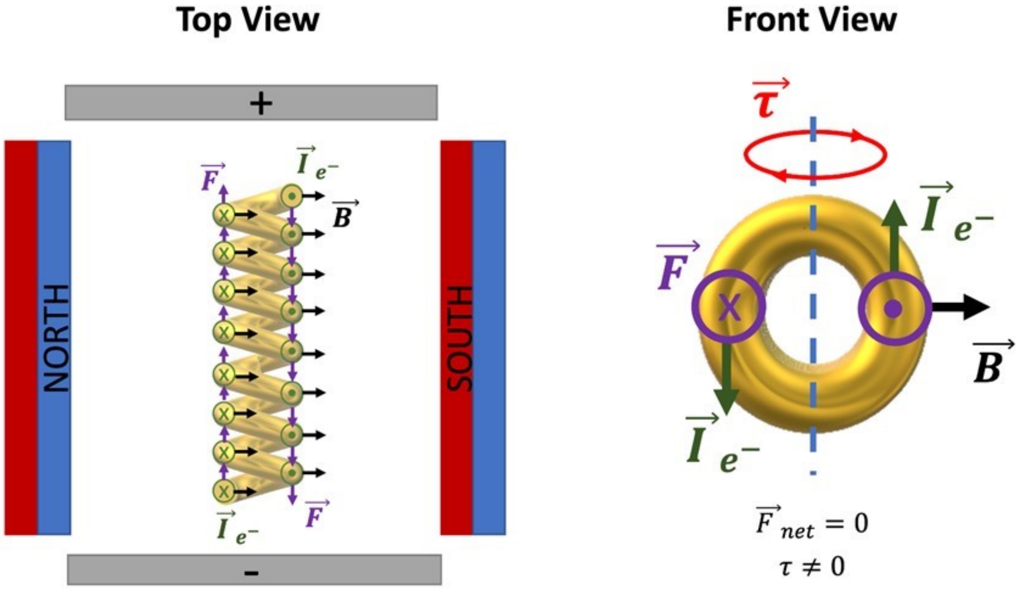

Figure S4. Top and front view of a solenoid swimmer placed in a uniform magnetic field ($\vec{B}$) with an external electric field driving a current of electrons ($\vec{I}_{e^-}$) though the solenoid and a representation of the resulting forces cancelling out producing a no net force ($\vec{F}_{net} = 0$) and the torque produced by the self-alignment of the magnetic dipole moment of the swimmer and the external magnetic field ($\vec{\tau} \neq 0$).

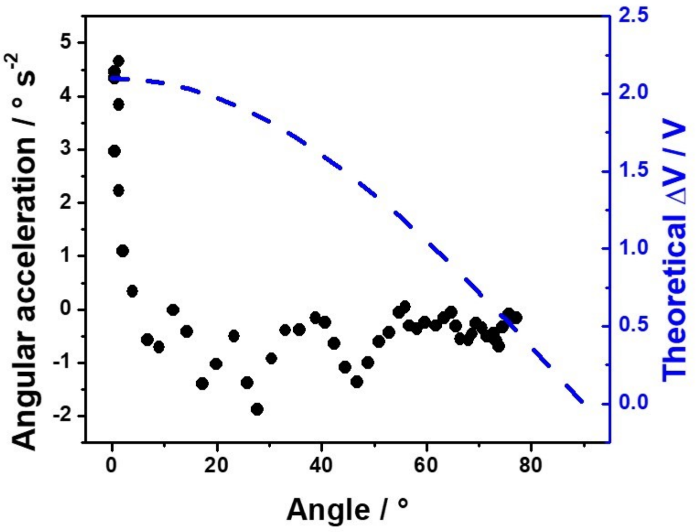

Figure S5. Angular acceleration (black curve) and theoretical polarization potential difference (blue curve) as a function of the angle of rotation, for a clockwise 5.7 mm long coil shaped BPE at a constant applied electric field (4.2 V cm^{-1}).

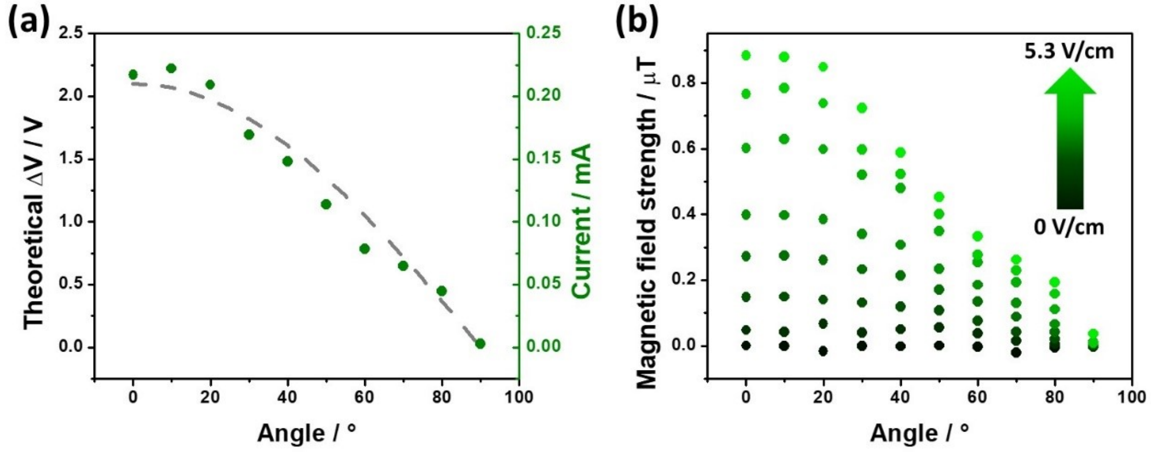

Figure S6. (a) Theoretical polarization potential (left axis grey dotted line) and amperometric measurements (right axis green dots) as a function of the angle of rotation, for a 5.7 mm long coiled shape BPE at a constant applied electric field (4.2 V cm^{-1}). (b) Average B_{swim} value estimated from amperometric measurements as a function of the angle of rotation at different applied electric fields (indicated in the figure) obtained in a 5 mM $LiClO_4$ /10 µM DBS solution containing a 1:1 ratio of HQ/Q (10:10 mM)

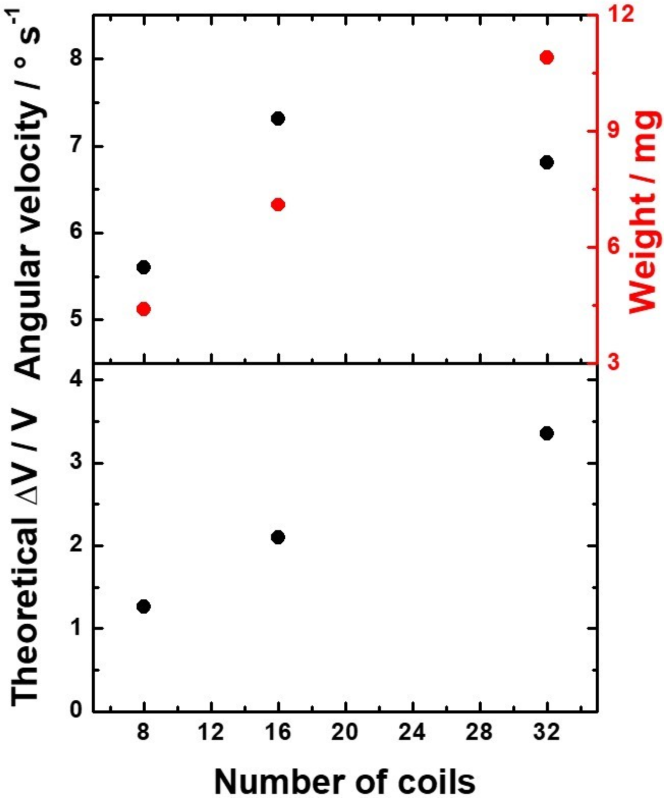

Figure S7. (Top) Maximum angular velocity (left axis black dots) and weight of the swimmer (right axis red dots) as a function of the number of coils, obtained in a 5 mM $LiClO_4$ /10 µM DBS solution containing a 1:1 ratio of HQ/Q (10:10 mM) at a constant applied electric field (4.2 V cm^{-1}). (Bottom) Theoretical polarization potential as a function of the number of coils at a constant applied electric field (4.2 V cm^{-1}).

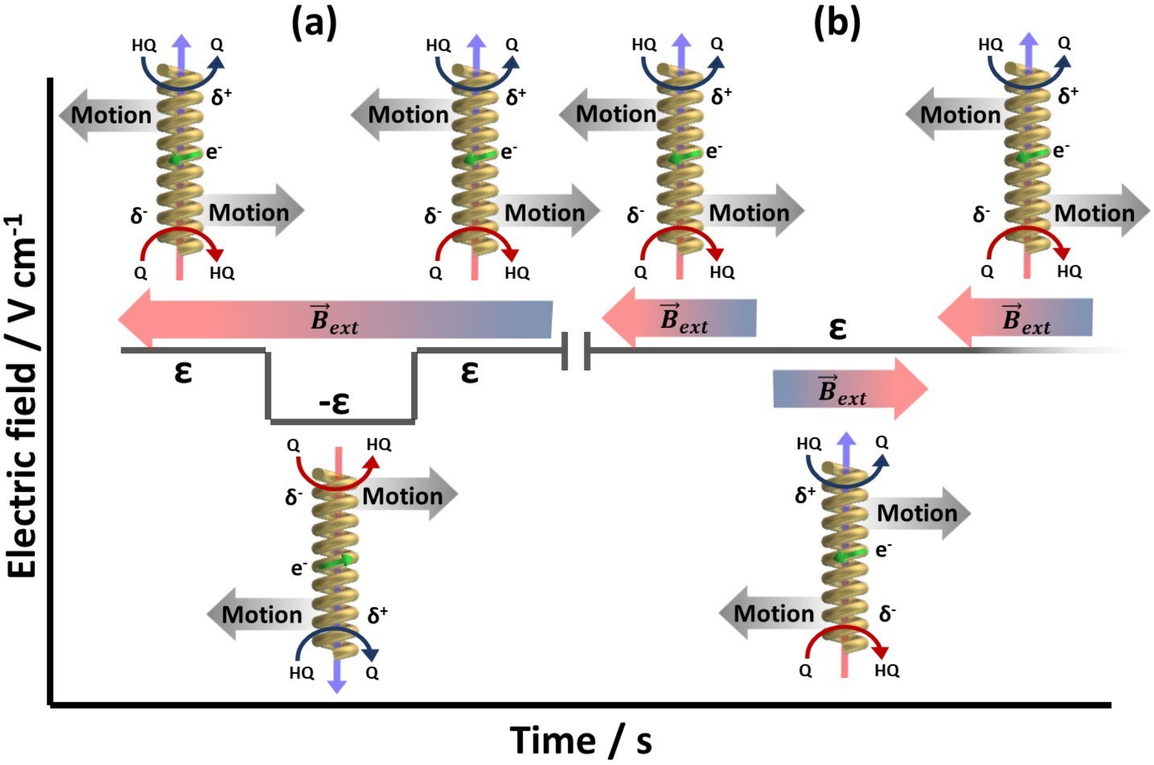

Figure S8. Schematic illustration of the oscillatory displacement triggered by using alternating electric (a) and magnetic fields (b), with a representation of the electric perturbation diagram, the orientation of the induced and external magnetic fields, the chemical reactions, the direction of the associated electron flow and the trajectory of the rotation.

Video S1. CW and CCW coil shaped Au/Ti BPEs moving at the air/water interface of a 5 mM $LiClO_4$ /10 µM DBS solution containing a 1:1 ratio of HQ/Q (10:10 mM) in the presence of an external magnetic field and a constant applied electric field (4.2 V cm^{-1}).

Video S2. Au wire moving at the air/water interface of a 5 mM $LiClO_4$ /10 µM DBS solution containing a 1:1 ratio of HQ/Q (10:10 mM) in the presence of an external magnetic field and a constant applied electric field (4.2 V cm^{-1}).

Video S3. CW coil shaped Au/Ti BPE moving at the air/water interface of a 5 mM $LiClO_4$ /10 µM DBS solution containing a 1:1 ratio of HQ/Q (10:10 mM) in the presence or absence of external electric and magnetic fields.

Video S4. CW coil shaped Au/Ti BPEs moving at the air/water interface of a 5 mM $LiClO_4$ /10 µM DBS solution containing a 1:1 ratio of HQ/Q (10:10 mM) in the presence of an external magnetic field for different applied electric field values.

Video S5. 8, 16 and 32 coils CW Au/Ti BPEs moving at the air/water interface of a 5 mM $LiClO_4$ /10 µM DBS solution containing a 1:1 ratio of HQ/Q (10:10 mM) in the presence of an external magnetic field at constant electric field (4.2 V cm^{-1}).

Video S6. CCW Au/Ti BPEs moving at the air/water interface of a 5 mM $LiClO_4$ /10 μM DBS solution in the presence of an external magnetic field for different applied electric field values.

Video S7. CW and CCW Au/Ti BPEs moving at the air/water interface of a 5 mM $LiClO_4$ /10 μM DBS solution containing a 1:1 ratio of HQ/Q (10:10 mM) in the presence of an alternating electric (3.1 V cm^{-1}) and magnetic field.
